# Editorial: Conflict Management and Trust Relationships in Organizations

**DOI:** 10.3389/fpsyg.2021.764332

**Published:** 2021-10-28

**Authors:** Patricia Elgoibar, Lourdes Munduate, Martin C. Euwema

**Affiliations:** ^1^Department of Business, University of Barcelona, Barcelona, Spain; ^2^Department of Social Psychology, Sevilla University, Seville, Spain; ^3^KU Leuven, Leuven, Belgium

**Keywords:** conflict, trust, cooperation, organizations, negotiation, mediation

## Background

Conflict management research recognizes that conflicts are inherently part of organizations and organizational dynamics. Different interests, different resources, and power relations drive both competition and conflict, while positive interdependences drive parties to cooperate. This is true for interpersonal relations at work, as well as for teams, departments, and at (inter)organizational level. Conflicts are not necessarily destructive, and often inevitable part of innovation and change, and constructive ways of conflict management are at the essence of modern and ethical organizational practices. Such recognizes the well-established scientific evidence that cooperation is at the heart of sustainable organizational success. Cooperative relations feed a joint problem-solving approach toward conflicts at all levels and is stimulated in organizational settings. One important antecedent as well as consequence of cooperation in organizations is mutual trust. The aim of this Research Topic is to promote research on constructive conflict management and trust in organizational life at all levels.

## Organization

The five empirical articles included in this Research Topic analyze the role of trust in organizational conflict in different contexts, such as collective negotiation, labor mediation, family firms, and religious organizations. They tackle different factors that can impact this relationship, such as emotions, trustworthiness, leadership, and conflict resolution methods. Diverse methodologies are used in this collection of papers—quantitative, qualitative and longitudinal—which contributes to the quality of understanding the link between trust and conflict management in organizations. In addition, these papers show results from data collected in different cultures, including Europe and Africa.

In the first paper by Elgoibar et al., titled “*Increasing integrative negotiation in European organizations through trustworthiness and trust*” the relation between trust and conflict is studied in a collective negotiation context, that is between managers and employee representatives (ERs). This paper, using a quantitative methodology, demonstrates the importance of both trustworthiness of ERs, and trust between management and ERs, to promote the impact of ERs on the outcomes of negotiation, particularly when it comes to integrative issues, compared to more traditional, distributive issues at the collective negotiation table.

The second paper by Kalter et al., titled “*A matter of feelings: Mediators' perceptions of emotion in hierarchical workplace conflicts”* puts its emphasis on the role of emotions in hierarchical conflict in a mediation context. A quantitative method is used to illustrate the differences in experienced positive and negative emotions of supervisors and subordinates during mediation. Mediators do not always perceive these emotions correctly, which impacts the effectiveness of the mediation.

The third paper, by Alvarado-Alvarez et al., titled “*Unraveling the role of shared vision and trust in constructive conflict management of family firms. An empirical study from a mixed methods approach,”* demonstrates the key role which trust plays to achieve constructive conflict handling, as well as the need for a shared vision in family firms in order to handle conflicts well.

The fourth paper, by Obi et al., titled “*Servant leadership stimulates spiritual wellbeing through team trust in a female religious context*” analyzes the role of servant leadership in fostering wellbeing in a unique setting, that is small convents, where sisters of African origin live and work together. The study demonstrates that servant leadership promotes trust among sisters, and reduces team conflict, thus contributing to (spiritual) wellbeing, operationalized through a new measure: “gifts and fruits from the Spirit.”

The fifth and last paper, by Estreder et al., titled “*The positive spiral between problem-solving management and trust: A study in organizations for individuals with intellectual disability”* uses a longitudinal approach to understand the dynamics in which problem-solving stimulates trust and vice-versa. The study is carried out in organizations for persons with intellectual disabilities. This specific context provides the opportunity to analyze long term relationships between professionals, disabled persons and their families, in which trust among them is crucial for the quality of life. The study tackles the debate about trust as antecedent as well as consequence of a problem-solving approach in conflict.

## Future Directions

Together, these five empirical studies all demonstrate in different ways the key role trust and trustworthiness plays in constructive conflict handling. Despite highly different contexts, relations, conflicts and cultures, and through diverse research methodologies, trust and trustworthiness contribute to long term relations, constructive conflict, wellbeing and also influence of parties. This indeed an upward spiral, where parties, being trustworthy, and handling conflict constructively, feed mutual trust, wellbeing and achieve better organizational outcomes. This process also holds, when conflictive issues grow, and stakes are high. Organizations and societies face immense challenges to cope with unprecedented environmental changes. The conflict potential rises on all levels. The outcomes of these different studies all point the only reasonable way forward: a shared vision in which all parties recognize the underlying positive interdependence, despite conflicts of interests, therefore see the need to cooperate. Trustworthiness as (global) partners, through demonstrated competence, integrity, benevolence and consistency, is needed more than ever. The academic challenge is, to demonstrate this “crude law of cooperation” also under severe conditions, and further test methods for constructive conflict management under high complex and dynamic circumstances.

## Author Contributions

All authors listed have made a substantial, direct and intellectual contribution to the work, and approved it for publication.

## Conflict of Interest

The authors declare that the research was conducted in the absence of any commercial or financial relationships that could be construed as a potential conflict of interest.

## Publisher's Note

All claims expressed in this article are solely those of the authors and do not necessarily represent those of their affiliated organizations, or those of the publisher, the editors and the reviewers. Any product that may be evaluated in this article, or claim that may be made by its manufacturer, is not guaranteed or endorsed by the publisher.

